# Editorial: Functional and Smart Biomaterials: Development and Application in Regenerative Medicine

**DOI:** 10.3389/fbioe.2022.920730

**Published:** 2022-05-17

**Authors:** Guicai Li, Hongbo Zhang

**Affiliations:** ^1^ Key Laboratory of Neuroregeneration of Jiangsu and Ministry of Education, Co-innovation Center of Neuroregeneration, NMPA Key Lab for Research and Evaluation of Tissue Engineering Technology Products, Nantong University, Nantong, China; ^2^ Pharmaceutical Sciences Laboratory, Åbo Akademi University, Turku, Finland; ^3^ Turku Bioscience Center, University of Turku and Åbo Akademi University, Turku, Finland

**Keywords:** functional biomaterials, smart biomaterials, development, application, regenerative medicine

The development of functional and smart biomaterials has emerged the multidisciplinary subjects including medicine, biology, physics, chemistry and materials science, etc., and is playing important role in clinical applications, e.g., facilitating wound healing and restoring other types of biological function (Freudenberg et al., 2016; Shodeinde et al., 2020; Najjari et al., 2022). However, the performance of these biomaterials still need to be further enhanced due to the complicated interactions with components of living systems and the unpredictable responses of body to biomaterials (Kowalski et al., 2018). Thus, a good design and development of functional and smart biomaterials will be a guarantee for their future clinical applications for improving patients’ health and life. Today, with the flourishing of biomaterials and regenerative medicine, more and more researcher are joining this field to study the functional and smart biomaterials for various regenerative medicine applications. To highlight the current progress, this Research Topic aims to bring together the latest exciting achievements referring to the development and application of functional and smart biomaterials on regenerative medicine.

Here, we collected a total of 16 papers, which present a broad range of the functional and smart biomaterials design, preparation, evaluation and application for various biological systems; and summarize the current progress of functional and smart biomaterials in regenerative medicine. The papers published in the present topic are briefly introduced below.

Dong et al. highlighted and summarized the various applied polymers in osteonecrosis therapy, then discussed the development of biofunctionalized composite polymers based on the polymers combined with different bioactive substances. Finally, the application of polymers in the treatment of osteonecrosis and future outlook are summarized. This review provided a comprehensive knowledge relevant to the application of polymers in the treatment of osteonecrosis and a meaningful theoretical basis further to advance the treatment of osteonecrosis with biomedical polymer materials.

The ideal orthopedic implant should possess both osteogenic and antibacterial properties and do proper assistance to *in situ* inflammatory cells for anti-microbe and tissue repair. However, aseptic loosening and peri-implant infection remain problems that may lead to implant removal eventually. Titanium and its alloys are dominant material for orthopedic/dental implants due to their stable chemical properties and good biocompatibility. In the review by Lu et al., an overview of the latest strategies to endow titanium implants with bio-function and anti-infection properties were overviewed. The methods for preparing efficient surfaces were stated and the insights into the interaction between the devices and the local biological environment were offered. In the end, the challenges in terms of stability and long-term performance were put forward, new substances or surface modification methods with antibacterial and bone-promoting properties need to be explored in the development of ideal materials for bone implantation.

Shu et al. introduced the advantages of hydrogel dressings and the treatment strategies for burns, ranging from external to clinical. They then discussed the development of new hydrogel dressings for wound healing along with skin regeneration, and the functional classifications of hydrogel dressings along with their clinical value for burns. To construct different functional hydrogel dressings according to the different stages of wound healing and ensure that the appropriate therapy is administered when appropriate in the treatment of burn wounds.

Xu et al. analyzed and summarized the construction methods, with or without cells, and repair effects of single layer scaffold and multi-layer scaffold for the treatment of esophageal cancer. The multilayer complex structure of the esophagus should be considered in the repair of the full-thickness or circumferential defect of the esophagus. Besides, the source of an ingenious design and maintenance of the bionic structure and bionic function are the research direction.

Wu et al. explored a novel method for constructing porous collagen membranes via the combined application of bioskiving and sonication. Tuning the power intensity was shown to modulate fibril orientation, and the porous membrane without denatured collagen could be obtained by a 20-min sonication treatment at 90 W. The prepared collagen membrane could also be further mineralized to enhance osteogenesis. Overall, this study offered a rapid and convenient approach for fabricating porous collagen membranes.

In another study, Wu et al. developed a sensitive, specific, and biocompatible integrin αvβ3-targeted superparamagnetic Fe_3_O_4_ nanoparticles (NPs) for the noninvasive magnetic resonance imaging (MRI) of integrin αvβ3. The results established the possibility of Fe_3_O_4_-RGD serving as a feasible MRI agent for the noninvasive diagnosis of IgA nephropathy.

Li et al. investigated the osteogenic differentiation of inducedpluripotent-stem-cell-derived mesenchymal stem cells (iPSC-MSCs) and bone regeneration capacities using N-acetyl cysteine (NAC)-loaded biomimetic nanofibers of hydroxyapatite/silk fibroin (HAp/SF), which demonstrated the promising potential for the use of NAC/HAp/SF for bone tissue engineering.

Zhao et al. synthesized a bioclickable mussel-derived peptide Azide-3,4-dihydroxy-Lphenylalanine (DOPA_4_) as a polyether ether ketone (PEEK) surface coating modifier and further combined bone morphogenetic protein two functional peptides (BMP2p) with a dibenzylcyclooctyne (DBCO) motif through bio-orthogonal reactions to obtain DOPA_4_@BMP2p-PEEK, which displayed excellent biocompatibility and osteogenic functions, thus offering insights to engineering surfaces of orthopedic implants.

Liu et al. reported a dual-response nano-carrier of glutathione and acid to achieve the rapid release of encapsulated drug and increase the effective drug concentration in the tumor. In this way, the nanocarrier degraded quickly, realizing the purpose of rapid drug release and efficient antitumor effects, thus showing better clinical application prospects.

Electrospinning is still the convenient and efficient method for constructing tissue engineered implants. Wang et al. prepared nanofibrous membranes with different gelatin/polycaprolactone mass ratios via electrospinning for preventing postoperative cardiac adhesion, also providing potential application for wound dressing and bone regeneration. Kong et al. developed a porous nerve decellularized matrix-chitosan (NDM-CS) scaffold with high antimicrobial activity and high biocompatibility using a one-step electrospinning method for neural tissue engineering.

Hong et al. synthesized the copolymer of 6-arm polyethylene glycol and heparin (PEG-Hep) and then immobilized it on the surface of chitosan (Chi)-modified magnesium alloy surface through electrostatic interaction, which was shown to improve the corrosion resistance and biocompatibility.

Luo et al. covalently immobilized multifunctional baicalin (BCL) onto the surface of the contact lens, thus improving the anti-inflammatory, anti-oxidative stress, and antibacterial capabilities, and displaying great application potential in the surface engineering of ophthalmic medical materials.

Chen et al. proposed a simple UV-photofunctionalization strategy to improve the hemocompatibility of Ag nanoparticles, which provided a new solution idea to improve the hemocompatibility of metal nanoparticles.

Zhang et al. constructed Ag-incorporated polydopamine/tannic acid coating on titanium substrate with improved hydrophilicity, good cytocompatibility, and antibacterial effectiveness, indicating the potential for surface modification of titanium implants.

In summary, the articles collected in this Research Topic demonstrate the development and application of functional and smart biomaterials in regenerative medicine. In prospectively, the functional and smart biomaterials will continue to expand their application and significance in the field of regenerative medicine and tissue engineering.
